# Sex difference in driving speed management: The mediation effect of impulse control

**DOI:** 10.1371/journal.pone.0288653

**Published:** 2023-07-17

**Authors:** Chunyang Pan, Jinfei Ma, Ye Li, Yang Lu, Lixin Shan, Ruosong Chang

**Affiliations:** 1 Liaoning Normal University, Dalian, Liaoning Province, China; 2 Dalian Technician College of Transportation, Dalian, Liaoning Province, China; Anhui Agricultural University, CHINA

## Abstract

Previous studies have shown that male drivers drive faster than female drivers, but there is no agreement on whether impulsivity could induce this sex difference, nor is there a cross-sectional comparison of the effects of different road environments. The purpose of this study was to verify whether impulsivity and impulse control could explain the sex differences in driving speed. A driving simulator study (study 1, N = 41) was performed to investigate whether there were sex differences in driving speeds in two road sections of different complexity, and a questionnaire survey (study 2, N = 163) was conducted to investigate the relationship between sex, impulsivity, impulse control and driving behavior of the participants. The results showed that male drivers drove faster on simple roads, but this difference did not show on complex roads. There were no sex differences in impulsivity traits, but male participants had significant lower levels of impulse control. The results also reveal a partial mediating role of impulse control in the relationship between sex and driving speed. These results suggest that impulse control can predict dangerous driving behaviors and is an important factor in explaining sex differences in driving speed.

## 1. Introduction

### 1.1 Driving speed and traffic safety

Driving is a complex and cognitively demanding activity, widely regarded as one of the most dangerous activities one can engage [1]. Statistics show that traffic accidents claim 1.35 million lives each year [2]. According to the National Bureau of Statistics of China, a total of 211,074 motor vehicle traffic accidents occurred nationwide in 2020, resulting in 55,000 deaths, 210,000 injuries and direct and indirect economic losses of 1.228 billion yuan [3]. Road traffic safety has significant public health and economic impact, and road safety should become an important research direction in the world.

Among the factors that can cause collisions, improper speed is considered to be one of the most important factors contributing to death and injury. In the US analysis of accidents involving young drivers in California and Maryland [4], it was estimated that driving too fast caused about 20% of accidents. Excessive speed not only reduces vehicle control, but also increases the risk of death in traffic accidents [5]. Researchers have found that increasing speed reduces the amount of time available to respond to sudden changes on the road, reduces the driver’s maneuverability and leads to greater stopping distances [6]. Moreover, many studies have shown that the increase of speed leads to an increase in the rate and severity of car accidents [7]. Bohlin [8] argue that there is some form of curvy relationship between injury and speed, with a much greater probability of serious injury or death at high impact speeds. To sum up, driving too fast will bring major traffic safety risks, and understanding the sources of drivers speeding behavior is a key link to formulate corresponding countermeasures.

### 1.2 The interaction between sex and road scenarios on driving speed

In past studies, researchers have suggested that sex is a predictor of risky driving behavior. After adjusting the mileage differences, the researcher found that the accident rate and accident injury degree caused by sex differences still existed [9], which was mainly manifested in speeding and violation. Hassan et al. [10] report that men are about 1.4 times more likely to be involved in speed-related crashes than women. Compared with female drivers, male drivers are more likely to be overconfident about their driving skills, and are more likely to perform speeding and drunk driving behaviors [11]. At the same time, male drivers also have more violations and receive more tickets due to improper speed control [12].

At the same time, sex difference has an interactive effect on drivers’ speeding behavior under different road conditions. Harré, Field and Kirkwood [13] found that men were significantly more likely than women to report engaging in unsafe driving behaviour and speeding on the open roads (like rural roads), while this difference was not evident on urban roads. Mischel [14] suggested that when manipulation was strong, it would cancel out the effect of any characteristics. Only when manipulation is weak or absent does a trait have a significant effect on one’s behavior. In this context, environmental variables must be taken into account to determine the extent to which the drivers’ personality is examined by the experimental settings. On more complex roads (such as urban roads), the mental load rises as vehicles and buildings occupy too much cognitive resources, and crowded lanes make overtaking impossible, so the differences in drivers’ personality traits are not obvious. On simpler roads, it is easier to observe the influence of drivers’ personality traits on driving behavior, as the influence of the environment is reduced. Some drivers drive faster on wider roads, possibly because of different levels of impulsivity and impulse control. Drivers with higher impulse control may be more willing to restrain their driving behavior and slow down, while drivers with lower impulse control may restrain themselves less and drive faster.

### 1.3 Effect of impulsivity on driving speed

In order to identify groups of people who are more likely to engage in risky road behavior, current studies either focus on one personality trait or compare the relative impact of several personality traits on dangerous driving behavior, with impulsivity being the most frequently examined variable. Impulsivity, as a personality trait, is defined as “the tendency to act without thought, consideration and assessment of consequences” [15]. A systematic review [16] in the field of traffic safety found that collisions and speeding were the most common impulsive-related issues. They also found that the relationship between impulsivity and drivers’ improper behavior was stronger than the correlation coefficient between impulsivity and accident. Therefore, these results appear to fit the general situational mediation model, in which personality traits are considered to influence the distal environment of accident involvement through the proximal environment involving driver factors. They also concluded a general contextual mediation model for personality, driving behavior and accidents based on previous research results.

Impulsivity was mostly measured using the BIS-11, and researchers came to different results. To be specific, no sex differences were found in the revision of the original questionnaire [17] or subsequent application in the Italian region [18]. That is, impulsivity did not explain the differences between the groups when they were grouped by sex. However, it was also found shown that male drivers are more functional impulsive and exhibit more impulsive or aggressive driving behaviors [19]. In view of the above disagreement, whether impulsivity show sex difference should be further verified. Furthermore, we should also consider the possibility that there are other potential factors that affect the influence of drivers’ impulsivity on specific driving behaviors.

### 1.4 The inhibitory effect of impulse control on speeding behavior

Another variable that is often mentioned in the area of traffic safety is impulse control. Impulse control is considered a dimension of executive function and is often measured using the EFI scale. Executive function refers to a series of top-down cognitive functions used when an individual cannot complete a task through automatic processing and must achieve shift or focus attention through cognitive efforts. Executive function includes a range of complex cognitive functions, such as working memory, inhibitory control, attentional control, cognitive flexibility, reasoning, planning, problem solving, etc. [20]. Poor impulse control can lead to problematic behaviors, such as swearing and anger, and in the field of traffic, problematic driving behaviors, such as aggressive driving and speeding. Ross et al. [21] measured drivers’ executive function through different behavioral experiments and proved that inhibition control was negatively correlated with speeding. Hayashi et al. [22] measured executive function and dangerous driving behavior of young drivers and found that the impulse control had a significant negative correlation with speeding. The lower the impulse control score, the more likely it was to speed. A review article [23] reported that impulse control enables drivers to effectively control speed, wear seat belts, avoid drunk driving, and reduce driving errors such as lane departures or other violations.

Unlike impulsivity, previous studies have generally considered impulse control to be sex-specific, with women scoring higher on inhibitory control than men [24], manifested by a greater ability to inhibit undesirable behaviors and control undesirable impulses. Thus, rather than two aspects of the same issue, impulsivity and impulse control may be two separate factors, and the level of impulse control affects the expression of impulsivity and leads to sex differences in risky driving behaviors (e.g., speeding).

### 1.5 Aim of the present study

This study consists of two parts. The first is the driving simulator experiment, which aims to investigate the sex differences in speed control under different road environments, and confirm whether impulsivity and impulse control of drivers show sex differences. The second study is a questionnaire survey aimed to verify whether impulsive personality and impulse control are mediating the influence of sex on drivers’ driving speed.

## 2. Study 1 sex difference in driving speed: A simualtor study

### 2.1 Methods

#### 2.1.1 Participants

Forty-one participants were recruited from Liaoning Normal University, including 17 males (accounting for 41.46%, average age = 24.41±3.79 years) and 24 females (58.54%, average age = 22±2.39 years). Each participant obtained a valid driver’s license and had good eyesight or corrected eyesight. All participants signed informed consent upon arrival at the experimental site. The experiment was approved by the Academic Ethics Committee of Liaoning Normal University. Each participant was assigned a participant number, and all data was collected anonymously.

#### 2.1.2 Materials

(1) Executive Function Index (EFI): The Executive Function Index (EFI) consists of 27 questions divided into five subscales, namely cognitive allocation, strategic planning, impulse control, motivational drive and empathy. The questionnaire is a 5-point Likert scale, ranging from 1 (never) to 5 (always), and the score of negative sequence item is 5 (never) to 1 (always). The total score ranges from 27 to 135. Higher score indicates higher level of the dimension and higher total score indicates better executive functions. EFI had acceptable intrinsic consistency (*α* = 0.79), and showed good content validity in clinical and neuroimaging studies.

(2) Barratt Impulsivity Scale-11(BIS-11): The Chinese version of the Barratt Impulsivity Scale-11(BIS-11) contains 30 questions divided into three dimensions, namely unplanned, action impulsivity and cognitive impulsivity. Each dimension contains 10 questions. Among them, action impulsivity is a positive item, corresponding to the score range of 1 (never) to 5 (always), while unplanned and cognitive impulsivity are both reverse items. The higher the total score is, the more impulsive the individual is. The internal consistency of BIS Chinese revision was good (*α* = 0.77–0.89), with retest reliability ICC = 0.69–0.89 [25].

(3) Driving simulator: Xuan Ai QJ-3A1 (small) driving simulator was used to simulate the driving task. The simulator has a visual Angle of 120°. It is composed of interactive visual system, simulated cockpit, electronic control system, customized software, accessory equipment and exterior accessories. It has video teaching, guided driving simulation exercises, interactive scene driving experiencing and accident tendency evaluation.

#### 2.1.3 Experimental design

A mixed experimental design of 2 (sex: male/female) × 2 (road complexity: simple/complex) was adopted. The intergroup variable was sex, including 17 male participants and 24 female participants. The variable within the group was road complexity. The simple section was a 4-lane expressway with 2 curves. The road has smooth surface, open vision and no obstacles. The complex section is a 2-lane mountainous road with more than 20 curves. The road is bumpy, the driver’s vision will be affected by the gradient fluctuations, and there are obstacles that drivers need to dodge at fixed locations.

The dependent variable is driving speed, including the average speed and maximum speed in simple and complex sections. The data are automatically recorded by the driving simulator. The maximum speed refers to the peak speed of the driver in the road section, which is an instant speed.

#### 2.1.4 Procedure

Before the formal experiment, the participants could take several minutes to be familiar with the driving simulator. The simulated driving task began when participants were ready, then participants were asked to complete two sections on different roads successively. In order to control the possible influence of the sequence of driving sections, dichotomy was adopted in this experiment. The first 21 participants arrived took part in the simple section first, while the rest were signed to the complex section first. In order to control the influence of fatigue, each driving section lasted 15 minutes, and there was a 5-minute break in between. Experiment was conducted from May 25, 2022 to June 20, 2022.

The instructions for the experiment read: “Driving on a highway (mountain road) will be simulated next, with a speed limit of 120km/h (60km/h on mountain road). Please obey the traffic regulations and complete the driving task according to your daily driving habits. During the simulated driving, there will be voice and graphic prompts for driving routes. Please follow the voice prompts for driving routes. If the engine stalls due to a collision or other unexpected events, please switch the gear back to neutral and start the engine again, then you can continue the driving task. The drive will last 15 minutes, and you will be informed when it’s complete. If you experience any dizziness or discomfort, please inform us immediately, and you may choose to terminate the experiment at any time”.

After reading the instructions, the questions of the subjects were answered to ensure that the subjects fully understood the instructions and the content of the driving simulation experiment. After the simulated driving task, the participants were asked to fill out the questionnaires.

### 2.2 Results

Table 1 shows the correlation between sex and scale scores and the maximum and average speed of two driving sections.

**Table 1:** Correlation between sex and speed management.

	1	2	3	4	5	6
1 sex	1					
2 Impulse control	-.29	1				
2 Maximum speed in simple road	.51**	-.48**	1			
3 Average speed in simple road	.50**	-.22	.70**	1		
4 Maximum speed in complex road	.09	-.27	.42**	.35*	1	
5 Average speed in complex road	.28	-.09	.37*	.56**	.70**	1

**. significant at 0.01 level (two-tailed)

*. significant at 0.05 level (two-tailed)

The results showed that sex was significantly correlated to the maximum speed and average speed of simple section. Average speed (*t* = 3.63, *p*<0.01) and maximum speed (*t* = 3.67, *p*<0.01) were significantly higher for male drivers in simple section than for female drivers, and no such difference was found in the complex section. Table 2 shows the correlation between impulse control and maximum and average vehicle speed in different sections. Impulse control was the only dimension related to driving speed, while scores on other dimensions had no significant correlation with driving speed. Impulse control was significantly negatively correlated with average speed on simple roads.

**Table 2:** Participants’ driving speed on simulator in different roads (in km/h).

Sex	Max speed in simple road	Avg. speed in simple road	Max speed in complex road	Avg. speed in complex road	Score on impulse control	Score on impulsiveness
Female	116.85±14.05	70.28±10.85	74.43±12.93	40.30±6.94	13.83±1.44	34.75±6.85
Male	136.25±16.57	83.63±12.34	76.79±15.03	44.11±6.10	13.00±1.32	34.06±6.08

**Fig 1:**
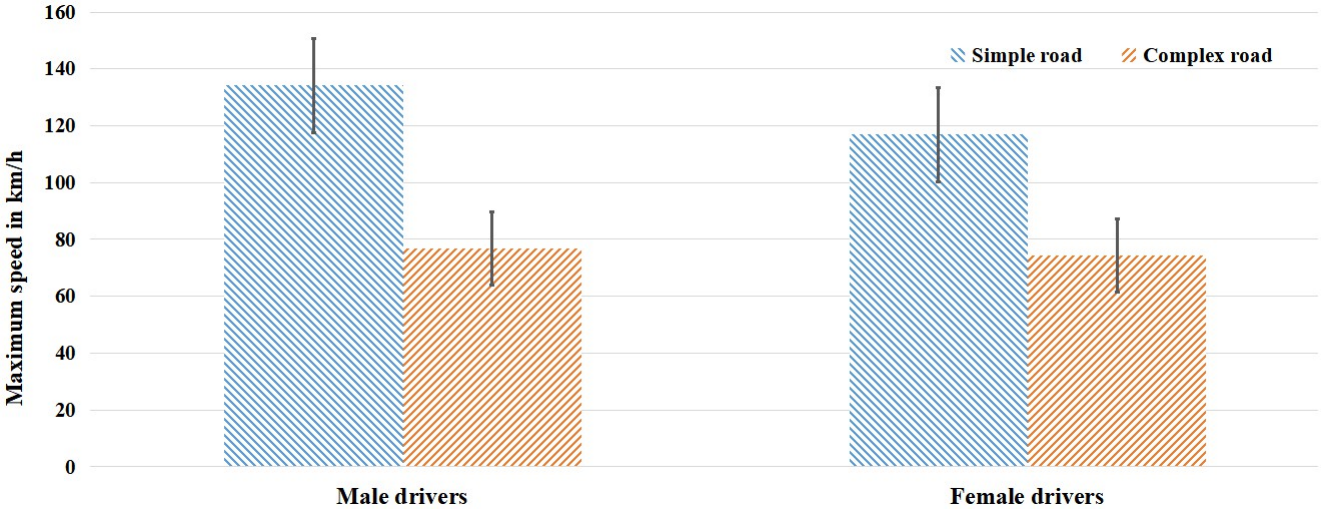
Maximum speed of drivers of different sex.

Table 2 shows the driving speed and scale score of drivers in simulated driving. The results showed that there was no significant difference in the total score of impulsivity scale, while the impulsivity control score of female drivers was slightly higher than that of male drivers (*p* = 0.066, significant margin). The main effect of sex and road complexity on the maximum speed of different complexity sections was significant, and the sex difference had significant interaction with the maximum speed of different sections, *F*_(1, 39)_ = 9.55, *p* < 0.01, *η*^2^ = 0.20. Simple effect analysis showed that the maximum speed of male drivers was significantly higher than that of female drivers, *F*_(1, 39)_ = 13.14, *p* < 0.01, *η*^2^ = 0.25, and the maximum speed of male drivers was significantly higher than that of female drivers. However, in the difficult sections, there is no significant sex difference in maximum speed, as shown in Fig 1.

**Fig 2:**
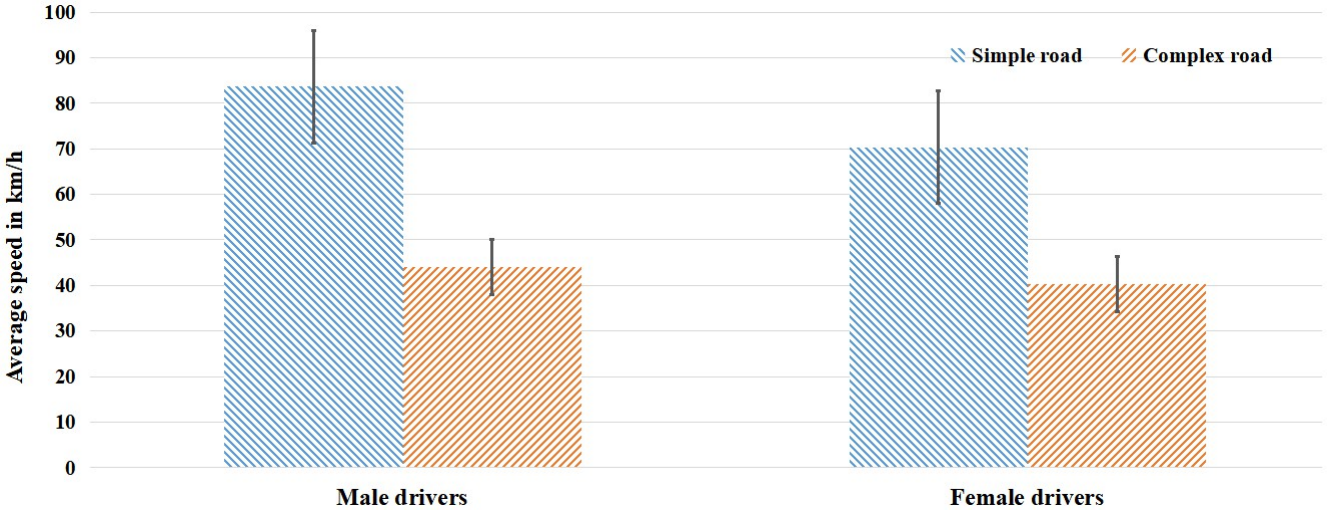
Average speed of drivers of different sex.

The main effects of sex and road complexity were significant, and the interaction between sex difference and the average speed of road complexity was significant, *F*_(1, 39)_ = 9.23, *p* < 0.01, *η*^2^ = 0.19. The simple effect analysis showed that the average speed in the simple section had significant sex difference, *F*_(1, 39)_ = 13.44, *p* < 0.01, *η*^2^ = 0.26. The average speed of male drivers in the simple section was significantly higher than that of female drivers. On the other hand, there is no significant sex difference in average speed in the complex section, as shown in Fig 2.

### 2.3 Discussion

Total score of BIS-11 and scores of each dimension did not show sex difference, suggesting that impulsivity could not explain why male drivers drove at faster speeds. In addition, the scores of impulse control between sex reached margin significance, which may be due to the limited sample size. We will conduct a questionnaire survey in a larger sample to verify sex differences in impulse control.

The maximum and average speed of male drivers on simple section is faster than that of female drivers. Previous studies have reported that men drive faster than women, possibly to relieve the sufferings in their lives, which in turn reduces anxiety and depression. Another given explanation is that male drivers are more likely to despise the possible consequences in the future, as they pay more attention to enjoyment in the current environment [26], but this assumption seems to be incomplete. If male drivers’ faster speeds simply rely on a stable personality trait, they would still drive faster than female drivers on complex roads, but the results found no such difference. Although male drivers have lower level of impulse control (marginal significant), they do not drive faster than female drivers on complex roads, indicating that they can adjust their driving behavior according to environmental requirements, rather than blindly driving fast. This emphasizes the importance of the influence of road environment on driver behavior. In terms of personality traits, Mischel [14] put forward a very important suggestion. When manipulation is strong, it cancels out any traits. Only when manipulation is weak or absent does a trait have a significant effect on behavior. The central processing unit (CPU) model in the self-control theory [27] also points out that when the external situation is too complex for an individual to deal with, the energy supply of cognitive resources will be short and fall into a state of self-depletion, and self-control cannot work effectively. In complex section, there are more road condition factors that need to be paid attention to. These complex and diverse road condition factors distract drivers’ attention from speed and instrument panel, so that they may adopt a faster way to move forward in the whole mountain road in the straight section of medium and high speed, so it is more likely to speed. Therefore, environment is a very important variable in predicting driver behavior, even over the influence of driver personality traits.

Finally, in order to examine sex differences in impulse control and whether they mediate the effect of sex differences on speed control, we will conduct a questionnaire study to examine a larger sample of drivers.

## 3 Study 2 examination of the mediating effects of impulse control on sex differences in speed control

### 3.1 Questionnaire distribution

Participants anonymously reported non-private personal information, including sex, age, miles driven and points on their driver’s license, according to the questionnaire, which was distributed through the website https://www.wjx.cn. They then completed the Executive Functioning Scale (EFI), the Barratt Impulse Scale (BIS-11) and the Dangerous Driving Behavior questionnaire DBQ. Questionnaires were collected from July 1, 2022 to August 18, 2022. After reviewing the content for completeness, participants received cash compensation. The research data were analyzed using IBM SPSS 20.0.

### 3.2 Materials

(1) Executive function index (EFI): See study 1.
(2) Barratt Impulsivity Scale-11 (BIS-11): See study 1.
(3) Dangerous driving behavior scale: The Chinese version of dangerous driving behavior scale consists three dimensions: overconfidence, speeding and traffic violation. It has 27 questions in total, with 9 questions for each dimension. The overconfidence dimension mainly asks whether the subjects will drive fast when others pay attention to or care about others. The speeding dimension mainly asks whether the subjects pursue speed in daily driving activities. The traffic violation dimension mainly asks whether the subjects often violate the rules and regulations in the daily driving tasks. It’s a 5-point Likert scale (1 for never and 5 for always). Higher score indicates stronger tendency to dangerous driving behaviors. This scale showed good internal consistency (α = 0.93) and was suitable for measuring the tendency of Chinese drivers to risky driving behavior.

### 3.3 Results

After collecting 177 questionnaires, 163 valid questionnaires were obtained after removing extreme data, which 73 were from male drivers (44.79%) and 90 were from female drivers (55.21%). In order to prevent the common method bias caused by the self-evaluation of questionnaire, Harman single factor test was performed. The first common factor explained 28.40% of the variance, which is less than 40%. Therefore, it could be considered that the self-evaluation of questionnaire in this study did not produce serious common method bias, and hence further data analysis would be valid.

See Table 3 for the correlation between sex and scores of each dimension of the 3 questionnaires. The results showed that sex was significantly correlated with the impulse control dimension of executive function, as well as with overconfidence and speeding in risky driving behaviors. In order to clarify the relationship between sex and various dimensions of executive function, independent sample t-test was conducted for executive function with sex as grouping variable. In the t-test of sex on each dimension of executive function, it was found that there were significant differences in impulse control between different sex, and the dimension of impulse control of female drivers was significantly higher than that of male drivers (*t* = 3.06, *p*<0.01).

**Table 3:** Correlation between sex, executive function, impulsive personality, and risky driving tendency.

	1	2	3	4	5	6	7	8	9	10	11	12	13	14
1 sex	1													
2 License points loss (last year)	.05	1												
3 Empathy	-.01	-.07	1											
4 Organization	.07	-.02	.39**	1										
5 Strategic planning	-.09	-.11	.22**	.41**	1									
6 Impulse control	-.24**	-.30**	.31**	.42**	.47**	1								
7 Motivational drive	.28**	.06	.29**	.45**	.34**	.10	1							
8 EF score total	.03	-.11	.65**	.80**	.73**	.63**	.61**	1						
9 Nonplanning impulsiveness	-.04	.11	-.33**	-.85**	-.44**	-.42**	-.43**	-.73**	1					
10 Motor impulsiveness	.06	.19*	-.20**	-.57**	-.52**	-.64**	-.21**	-.62**	.61**	1				
11 Attentional impulsiveness	-.14	.05	-.36**	-.67**	-.41**	-.31**	-.53**	-.67**	.74**	.51**	1			
12 Over-confident	.25**	.31**	-.34**	-.35**	-.45**	-.68**	-.15	-.55**	.39**	.55**	.28**	1		
13 Speeding	.27**	.22**	-.25**	-.45**	-.42**	-.63**	-.18*	-.55**	.46**	.53**	.32**	.77**	1	
14 Violation	.14	.18*	-.29**	-.31**	-.32**	-.56**	-.23**	-.47**	.32**	.41**	.27**	.64**	.74**	1

*: significant at 0.05 level(two-tailed)

**: significant at 0.05 level(two-tailed)

The dimensions of impulse control, cognitive allocation, strategic planning and empathy are significantly negatively correlated with the dimensions of tendency to dangerous driving behavior, and the dimensions of motivation are significantly negatively correlated with speeding and other violation of regulations. In general, executive function is significantly correlated with dangerous driving behavior. All dimensions of the tendency to dangerous driving behavior were negatively correlated with the driving license points deducted by the participants in the past year, indicating that the revised dangerous driving behavior scale has good ecological validity.

**Fig 3:**
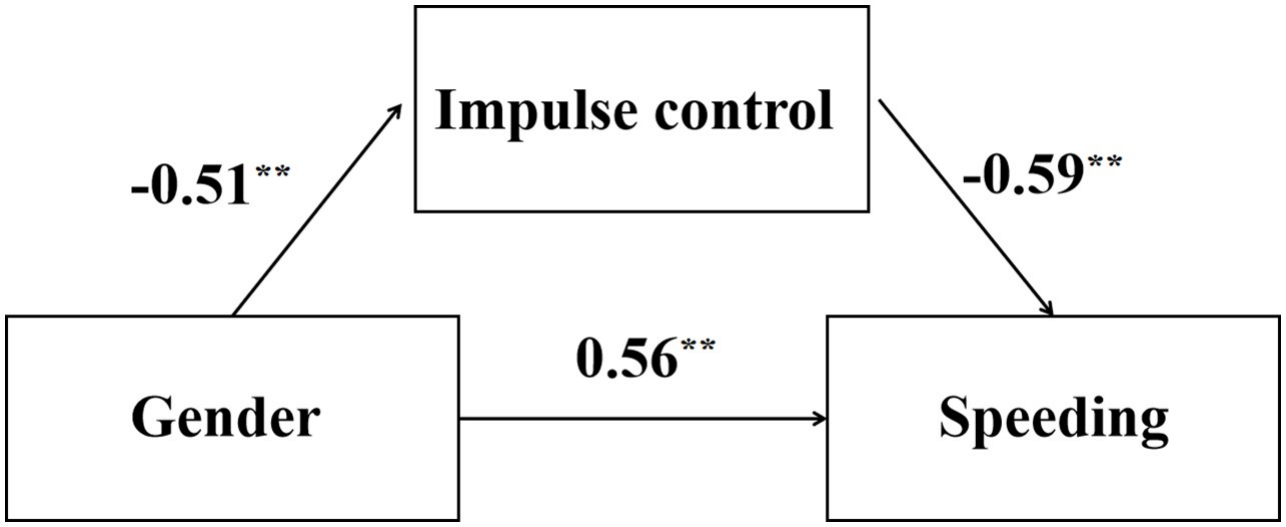
Standardized regression coefficients in the mediation of sex by impulse control in predicting speeding tendency. **: *p*<0.01.

Using Process v3.4 (Model 4) developed by Hayes, we tested the mediating effect of impulse control on the relationship between sex and speeding tendency, controlling for age (see Table 4). The results showed that sex had a significant predictive effect on speeding tendency. In addition, both the upper and lower limits of the bootup 95% confidence intervals for the direct effect of sex on speeding tendency and the mediating effect of impulse control are non-zero, suggesting that sex not only directly predict speeding tendency, but also predict speeding tendency through the mediating effect of impulse control (see Fig 3).

**Table 4:** Decomposition table of the total effect, direct effect, and mediating effect.

	Effect	BootSE	BootLLCI	BootULCI	Effect ratio
Impulse control	1.00	0.35	0.37	1.75	54.28%
Direct effect	0.83	0.42	0.02	1.64	45.72%
Total effect	1.83	0.51	0.83	2.85	

### 3.4 Discussion

Sex is highly correlated with the tendency to risky driving behavior, with female drivers having lower overconfidence and speeding tendency than male drivers, a result consistent with previous studies. Previous studies have shown that female drivers are more willing to obey the speed limit. In contrast, male drivers believe they can drive safely even when drunk or tired [28].

There were no sex differences in the scores of BIS-11, but the level of impulse control in males was significantly lower than that in females. These results were consistent with previous studies [17, 18], which suggests that impulsivity and impulse control have independent effects on driver behavior. BIS-11 measures personality traits, which can be influenced more by cultural and social factors, while EFI results partly reflect biological levels of development. At the very beginning of the formulation of EFI [29], the score was intended to reflect the physiological measurement results. The results showed that in the factor analysis, the three factors constituted by the five dimensions of the scale were in line with the expectation of Cummings [30] to identify dorsolateral, orbitofrontal and medial prefrontal circuits respectively. The factors of the scale were parallel to the functional organization of the prefrontal circuit. Whether there is a sex difference in scores on the two scales may be caused by the difference in purpose of the two measurements. In a society with decent sex equality, there is no difference in life experience between men and women and it is reasonable that they form alike personality traits, but the biological differences cannot be ignored. Previous studies have found that females perform better in response inhibition [31], which may be due to the larger volume of the orbital prefrontal cortex in females than in males [32], so females perform better in related functions that are more dependent on the orbitofrontal cortex (such as impulse control). In context of traffic safety, this can be seen as a natural advantage, and male drivers may need extra training to achieve the same level of impulse control as female drivers naturally do.

In addition, our results found that impulse control partially mediates the effect of sex on drivers’ tendency to speed. The statistical results showed that the impulse control score of male drivers was significantly lower than that of female drivers, and the impulse control score was significantly negatively correlated with the tendency to speeding, suggesting that the difference in impulse control level was an important source of the driving behavior differences between male and female drivers. In practical application, whether screening or training, attention should be paid to the level of impulse control of male drivers.

## 4 General discussion

The results show that there is no sex difference between drivers’ maximum speed and average speed on complex roads, possibly because complex environment limits the expression of drivers’ individual differences. On simple roads, however, the effects of the environment are diminished and sex differences are evident, with men driving significantly faster than women. Driving, as Taylor [33] points out, is a self-paced task, controlled by the level of emotional tension or anxiety the driver wishes to tolerate. Previous interventions to try to reduce the number of accidents did not bring satisfying changes because they ignored drivers’ subjective factors, since drivers do not always perform at their best. Taylor suggested that making the roads smoother, more visible would make drivers feel safer and perceive lower level of risk. Since when drivers feel safe, they don’t necessarily of so these settings cannot produce promising effect of reducing accidents as intended. On the one hand, the results highlight the importance of environmental factors when examining driver behavior. When trying to reduce road accidents, it is important to have settings that allow drivers to keep their vigilance within reasonable limits than to create more comfortable conditions for drivers. On the other hand, the results suggest that interventions to reduce speeding should mainly focus on male drivers on simple roads.

Impulse control is an important factor in explaining sex differences in speed control. Impulsivity is defined as the tendency to respond quickly and unanticipated to internal or external stimuli without regard to the negative consequences of those responses for the impulsive individual or others [34]. Poor impulse control can lead to problematic behaviors, such as swearing and anger. In the field of traffic safety, aggressive driving and speeding are examples of the problematic driving behaviors researchers concern. Most previous studies regard sex and impulse control (or impulsivity) as two separate variables, and impulse control studies mainly focus on clinical populations, such as patients with Parkinson’s disease [35] and patients with addiction [36]. Not enough attention is paid to the non-clinical population. The results of the present study reveal sex differences in impulse control in the non-clinical population. The impulse control level of male drivers is significantly lower than that of female drivers, and the impulse control level is negatively correlated with the tendency to speeding. Finally, impulse control partially mediated the effect of sex on speeding behavior.

According to Roberts and Yoon [37], we can identify at least four distinct areas or units of analysis in personality psychology: personality traits, motives, skills/abilities, and narrative identity. For example, social identity theory [38] states that people of different sex classify themselves as “socially acceptable male or female” and that they gradually approach the general characteristics of the group through identity mechanisms. As individuals become aware of the presence of others, they receive subtle external feedback, then males and females grow into socially acceptable men and women. This categorization-recognisation-comparison mechanism continues throughout life. In daily life, men are expected to be feisty, aggressive, and confrontational, while women are expected to be gentle and shy, which can be interpreted as a difference in narrative identity between men and women. At the same time, the social environment can also shape an individual’s ability, such as impulse control. Men undertake less pressure to control their behavior, as they are encouraged to compete, and they show lower levels of impulse control as a result. Male drivers were more likely than female drivers to view driving as an “exciting and competitive activity”, and are more likely to driver fast or take other risks to show their courage and fearlessness while driving. They are more likely to view other drivers on the road as competitors [39] and therefore speed more frequently.

On the one hand, impulse control partially mediates the relationship between sex and driving speed, and we should continue to explore other influencing factors to supplement the mechanism model of sex’s influence on speeding behavior. On the other hand, different aspects of personality may interact with each other, and more multi-factor researches are required to understand the differences in driver behavior.

Finally, the social factors that contribute to individual personality differences may vary from culture to culture, and we recommend that researchers look carefully at the conclusions drawn by researchers from different countries or regions.

## 5 Conclusions

The sex difference in driving speed was different in roads with different complexity. On simple roads, male drivers drove significantly faster than female drivers, but there was no sex difference in driving speed on complex roads. There was no significant difference in impulsivity between male and female drivers, but there was a sex difference in impulse control, with female drivers reporting significantly higher levels of impulse control, less speeding behavior, and less tendency for other risky driving behaviors. Finally, impulse control partially mediates the relationship between sex and driving speed.

## 6 Future directions and implications

Many previous studies have shown that male drivers are more likely to speed, meaning that interventions aimed at reduce speeding behaviors should probably mainly focus on male drivers. As a result, understanding the sex difference in driving behaviors becomes the key point. The results of the present study confirm that the difference in impulse control is one of the source of sex difference in drivers’ speed control, and difference in impulse control has an interaction with road complexity. This tells us that specific scenarios and drivers’ sex must be considered when implementing interventions aimed at improving road traffic safety. For example, in order to reduce speeding behaviors on simple roads (like highways), we should focus on safety education for male drivers like adding relevant courses to driver training programs. In addition, since speeding drivers are more likely to have low impulse control levels, impulse control training programs, in addition to fines or other punishments, may help reduce future speeding behaviors.

In future studies, we can continue to investigate the formation mechanism of impulse control and relevant interventions. On the other hand, we also need to conduct more multi-factor studies to explore the possible interaction between factors that affect drivers’ driving behaviors, so as to have a more complete understanding of the individual differences of drivers’ driving behaviors.
